# Evaluation of color stability in single-shade composite resins using spectrophotometer and cross-polarized mobile photography

**DOI:** 10.1186/s12903-025-05651-w

**Published:** 2025-02-22

**Authors:** Hatice Tepe, Ozge Celiksoz, Batu Can Yaman

**Affiliations:** Faculty of Dentistry, Department of Restorative Dentistry, Eskisehir Osmangazi University, Eskisehir, Turkey

**Keywords:** Single-shade composite resin, Color stability, Spectrophotometer, Cross-polarized photography, Aesthetic dentistry, Thermal cycling, Staining, Brushing

## Abstract

**Background:**

This study aimed to evaluate the long-term color stability of single-shade composite resins when exposed to simulated intraoral conditions, including staining, brushing, and thermal cycling. Additionally, the study compared the performance of spectrophotometry and cross-polarized (CP) photography in detecting color changes, focusing on their applicability for both clinical and research purposes.

**Materials and methods:**

The study employed five single-shade composite resins (Omnichroma; OMN, Zenchroma; ZNC, Vittra APS Unique; VTR, Charisma One; CHR, and Essentia Universal; ESU) and one multi-shade composite resin (Filtek Z550: FLT) for testing. The specimens were subjected to thermal cycling, staining, and brushing to simulate intraoral conditions. Color measurements were conducted using a spectrophotometer and CP photography at the initial baseline (t_0_) and following each subsequent procedure 10-day (t_1_) and 1-year simulated staining, brushing and thermal cycling (t_2_). Color differences (ΔE_1_, ΔE_2_) were calculated using the CIEDE2000 formula. Statistical analyses included Shapiro-Wilk for normality, ANOVA and paired t-tests for parametric data, and Kruskal-Wallis and Wilcoxon tests for non-parametric data. Method consistency was evaluated with the Intra-Class Correlation Coefficient (ICC) (*p* < 0.05).

**Results:**

All composite groups demonstrated statistically significant color changes following the simulated conditions (*p* < 0.001). The CHR group exhibited the highest values for both ΔE_1_ and ΔE_2_, indicating the greatest degree of discoloration. The FLT and ZNC groups exhibited the lowest ΔE values across methods at various time points. A positive correlation was identified between spectrophotometer and CP photography measurements for all parameters (*p* < 0.001), although the correlation for ΔE_1_ and ΔE_2_ was less robust.

**Conclusions:**

Single-shade composites display a high susceptibility to significant discoloration when subjected to simulated intraoral conditions, with CHR exhibiting the most pronounced alterations. Both spectrophotometry and CP photography were effective in assessing color stability, with CP photography offering a practical alternative for clinical settings. These findings offer insight into the aesthetic durability of single-shade composites and underscore the importance of long-term evaluations.

## Introduction

Composite resins are widely used in restorative dentistry for both anterior and posterior restorations due to their optical and mechanical properties that closely mimic natural tooth structure. Their high aesthetic potential and versatility have made them essential materials in modern clinical practice [1]. However, traditional composite resins often require a precise shade-matching process to achieve an optimal aesthetic result, which can be time-consuming and technique-sensitive [2]. To address this issue, single-shade composite resins have been developed, offering a universal shade that adapts to various tooth colors through the chameleon effect. These materials are designed to adapt to a wide range of tooth shades using a single universal shade, simplifying the restoration process while maintaining aesthetic outcomes [3]. This eliminates the need for multiple shades and reduces chair time.

Omnichroma (OMN; Tokuyama, Japan) is a universal single-shade composite resin, designed with a pigment-free formulation. This suprananofilled composite employs “smart chromatic technology,” which enables it to respond to specific light frequencies and reflect wavelengths within the tooth color spectrum, ensuring a natural color match [4]. Charisma Diamond One (CHR; Kulzer, Germany) is a nanohybrid composite that utilizes the “adaptive light matching” concept, where the shade of the restoration is determined by absorbing the wavelengths reflected from the surrounding tooth structure [5]. Vittra APS Unique (VTR; FGM, Brazil) is another nanohybrid composite that features a “blending effect,” replicating the color of the underlying tooth structure during polymerization. It incorporates Advanced Polymerization System (APS) technology, which combines multiple photoinitiators to enhance light-curing efficiency, resulting in improved polymerization conversion rates and mechanical properties [6]. Zenchroma (ZNC; President Dental, Germany) features ultra-fine radiopaque filler particles, enabling it to mimic the color tones of surrounding tissues, thereby supporting a natural appearance and simplifying color selection for clinicians [7]. Essentia Universal (ESU; GC Corporation, Japan), known for its wide color matching ability, provides a practical alternative to multi-shade composites, particularly for aesthetic restorations. Together, these materials offer significant advantages in terms of aesthetics and ease of use due to their single-shade design.

These materials leverage the chameleon effect, which allows them to blend with the surrounding tooth structure by adjusting translucency and light reflection [3, 8]. Single-shade composites offer significant advantages in terms of handling and clinical efficiency, especially in cases where precise shade matching is difficult, such as posterior restorations or discolored teeth [3]. However, despite these benefits, questions remain regarding their long-term color stability, particularly under the influence of staining agents and aging processes. Understanding the performance of single-shade composites in such conditions is critical for determining their overall clinical effectiveness.

With the increasing demand for aesthetics, the color stability of composite resins has become a critical area of research in restorative dentistry. The long-term resistance of composite materials to color changes plays a key role in the aesthetic success of restorations [9]. Therefore, evaluating the color stability of newly developed composite resins in vitro is essential for predicting their clinical durability. These studies simulate external factors under controlled conditions, helping to foresee potential issues that may arise in practice. Color changes are often triggered by both extrinsic and intrinsic factors, and in vitro tests provide valuable insights into these mechanisms [10]. 

Composite resins are continuously exposed to dietary staining agents and daily brushing in the oral environment. Pigmented substances such as coffee, tea, and wine can cause surface discoloration in composite resins [10]. While brushing can remove these surface stains, it can also lead to surface abrasion and contour loss, increasing roughness and promoting further staining [11]. Furthermore, thermal cycling, which simulates the temperature fluctuations experienced in the oral cavity, induces internal stress in composite resins, leading to the formation of microcracks and increased water absorption [12]. These factors degrade both the mechanical and aesthetic properties of composite resins over time [11, 12]. 

Color matching technologies have been developed to enhance the accuracy of shade selection, improve communication between clinicians and technicians, and better replicate the natural characteristics of teeth [13]. In dentistry, visual color selection is commonly performed using shade guides, a widely used but highly subjective technique. However, current commercial shade guides often lack standardization in material composition and specimen design, which can affect color matching accuracy. It has been suggested that an ideal shade guide should be made from the same composite materials used clinically, with standardized enamel and dentin layering to increase color matching reliability [14]. Instrumental methods, on the other hand, make the color selection process faster and more objective [15]. These methods include spectrophotometers, colorimeters, digital photography, and intraoral scanners [16, 17]. Research indicates that dental spectrophotometers provide the highest precision and accuracy in color matching [18]. Additionally, the use of cross-polarizing (CP) filters reduces unwanted reflections by minimizing flash-induced light reflection during photography [19]. Devices such as Smile Lite MDP (Smile Line, Switzerland) function as compact, portable devices for intraoral photography, enabling the use of various lighting conditions and facilitating the capture of high-quality intraoral images using smartphones. Despite the promise of these technologies, there is a scarcity of studies that integrate both spectrophotometer and mobile dental photography to thoroughly assess the color stability of composite resins, underscoring the need for more comprehensive research in this area [13, 20–22]. 

The purpose of this study was to evaluate the color stability of single and multi-shade composite resins subjected to staining, brushing, and thermal cycling using both spectrophotometer and CP photography. In addition, the agreement between the color measurements obtained by both methods was evaluated. Unlike previous studies that focused primarily on initial shade matching, this study uniquely evaluates the long-term shade stability of these materials under simulated intraoral conditions. The results underscore the susceptibility of these materials to discoloration and highlight the importance of material selection in maintaining esthetic outcomes in restorative dentistry. In addition, CP photography has the potential to serve as a practical and accessible tool for clinical shade assessment, providing reliable results comparable to those obtained by spectrophotometry.

The null hypotheses of this study were as follows: The color changes observed in the composite resins after staining, brushing, and thermal cycling would not be statistically significant for either single-shade or multi-shade composites. Additionally, no significant correlation would be found between the color measurements obtained from spectrophotometry and CP photography.

## Materials and methods

### Experimental study design

**Fig. 1:**
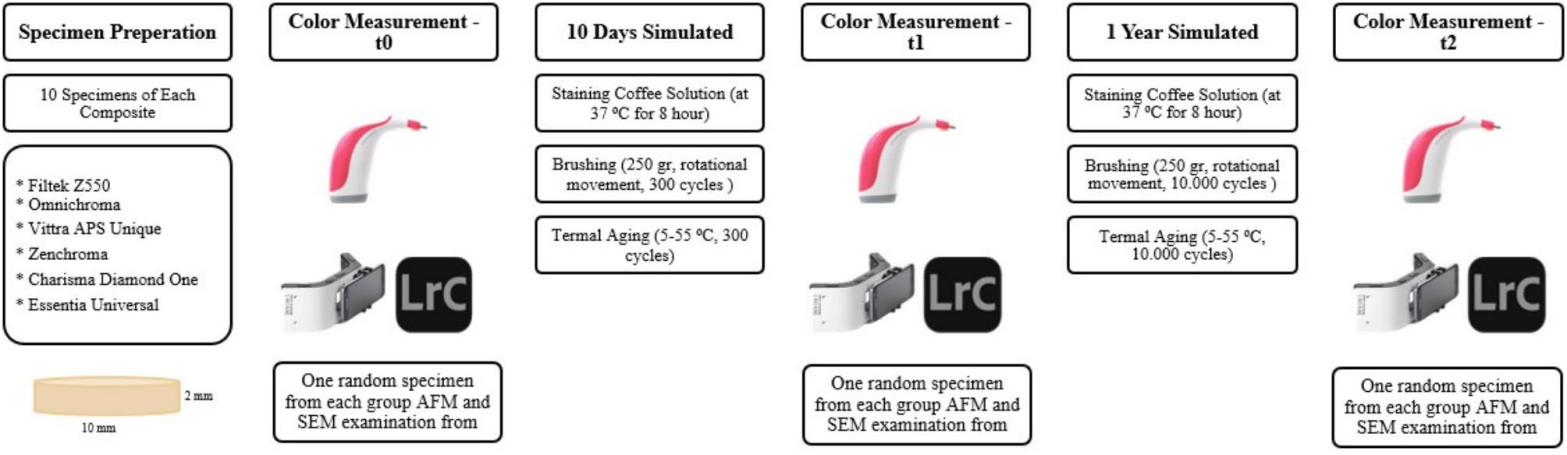
Flow chart of the experimental design

This study analyzed five single-shade composite resins: OMN, VTR, ZNC, CHR and ESU and one multi-shade composite resins, Filtek Z550 (3M ESPE, USA) (FLT). The categories, manufacturers, lot numbers, and compositions of the composite resins are presented in Table 1; Fig. 1 illustrates the study design, showing the flow of specimens through the various stages of the experiment. All specimens underwent staining, brushing, and thermal cycling processes.

**Table 1:** Materials used in the study

Material	Code	Material type	Composition	Filler Content		Filler size	Shade	Manufacturer	Lot number
				(wt %)	(vol %)				
Filtek Z550	FLT	Nanohybrid	BIS-GMA UDMA BIS-EMA PEGDMA TEGDMA	81.8%	67.8%	0.01–3.5 μm	A2	3M ESPE, USA	NC45123
Omnichroma	OMN	Nanofilled	Spherical silica-zirconia filler composite filler 1,6-bis(methacryl-ethyloxycarbonylamino) trimethyl hexane UDMA TEGDMA Mequinol Dibutyl hydroxyl toluene UV absorber.	79%	68%	0.3 μm	Single Shade Universal	Tokuyama, Japan	044EZ0
Vittra APS Unique	VTR	Nanohybrid	UDMA TEGDMA Photo initiator composition (APS), Co-initiators Stabilizers Silane. Boron-aluminum-silicate glass	72-80%	52-60%	0.2 μm	Single Shade Universal	FGM, Brazil	230,921
Zenchroma	ZNC	Microhybrid	Glass powder Silicon dioxide UDMA Bis-GMA, TEGDMA	75%	53%	0.005–3.0 μm	Single Shade Universal	President Dental, Germany	2,022,003,395
Charisma Diamond One	CHR	Nanohybrid	Barium Aluminum Boro Fluor Silicate Glass TCD-Urethane acrylate Silica UDMA TEGDMA Titanium Dioxide, Fluorescent Pigments Metallic Oxide Pigments Organic Pigments Aminobenzoic acid ester BHT Camphorquinone	81%	64%	0.05–20 μm	Single Shade Universal	Kulzer, Germany	K010025
Essentia Universal	ESU	Microhybrid	UDMA Bis-MEPP Bis-EMA Bis-GMA TEGDMA PPF Strontium glass Lanthanide fluoride Fumed silica FAISi Glass	91%	61%	0.1 μm	Single Shade Universal	GC Corp, Japan	2,007,231

Color measurements were performed using two methods: a spectrophotometer (Vita Easyshade V, Vita Zahnfabrik, Germany) and a software program (Adobe Photoshop Lightroom Classic V.12.0.1, Adobe Inc., USA). The color analyses were based on CP photographs captured with a mobile dental photography device (Smile-Lite MDP, Smile Line, Switzerland) and a smartphone (Xiaomi 11T Pro, Xiaomi Corporation, China) at three time points: baseline (t_0_) and following each subsequent procedure: 10-day simulated staining, brushing, and thermal cycling (t_1_) and 1-year simulated staining, brushing, and thermal cycling (t_2_).

### Specimens size calculation

The specimen size was calculated using G*Power statistical software. Based on a confidence level of 95% (1-α), a test power of 95% (1-β), and an effect size (f) of 0.655, the total specimen size required for one-way analysis of variance (ANOVA) was determined to be 54, with 9 specimens in each group [23]. To account for potential specimen loss, the study was designed with 10 specimens allocated to each group.

### Specimens preparation

A total of 60 specimens (*n* = 10 per group) were prepared using silicone molds with dimensions of 10 × 2 mm [10]. After placing the composite resin into the molds with a slight overflow, a mylar strip and microscope slide were placed on the upper surfaces, and the material was polymerized for 10 s using a curing light (D-Light Pro, GC Corporation, Japan). The slide was then removed, and polymerization continued by applying the curing light for an additional 10 s over the mylar strip, following the manufacturer’s instructions. The same curing light was used for all polymerization steps, and the light output was periodically checked with a radiometer (Woodpecker LED-F, Woodpecker Medical Instrument Co., China) to ensure an intensity of at least 1000 mW/cm² throughout specimen preparation.

Following polymerization, each specimen was polished using a polishing disc system (Optidisc, Kerr Corporation, USA), progressing from extra-coarse to extra-fine at a speed of 10,000 rpm, with each step lasting 10 s. A new disc was used for each specimen. The specimens were then rinsed with water for 10 s to remove any debris from the surface, and stored in distilled water at 37 °C in an incubator for 24 h post-polymerization [10]. All procedures were performed by a single operator.

To control the effect of pressure on polishing accuracy, the initial and final thickness of each specimen was measured three times by the same operator using an industrial-type digital caliper (0.01 mm accuracy) with a 0–150 mm measuring range [24]. 

### Color assessment and measurement

Color assessments were conducted using two distinct methods at three different time points: baseline (t_0_), after 10 days (t_1_), and after 1 year (t_2_) simulation. These methods aimed to capture both objective spectrophotometric data and photographic evaluations for comprehensive color analysis.

The first method involved measuring the color of the specimens using a digital spectrophotometer (Vita Easyshade V, Vita Zahnfabrik, Germany) to obtain L*, a*, and b* color coordinates. The spectrophotometer was calibrated before each set of measurements to ensure accuracy. Color measurements were performed three times at the center of each specimen, and the average values of the L*, a*, and b* parameters were recorded for further analysis. To standardize the measurements, an 18% grey card (L*=50, a*=0, b*=0; JJC Photography Equipment Co. Ltd, China) was used as a reference to ensure consistent lighting and white balance during the spectrophotometric measurements.

**Fig. 2:**
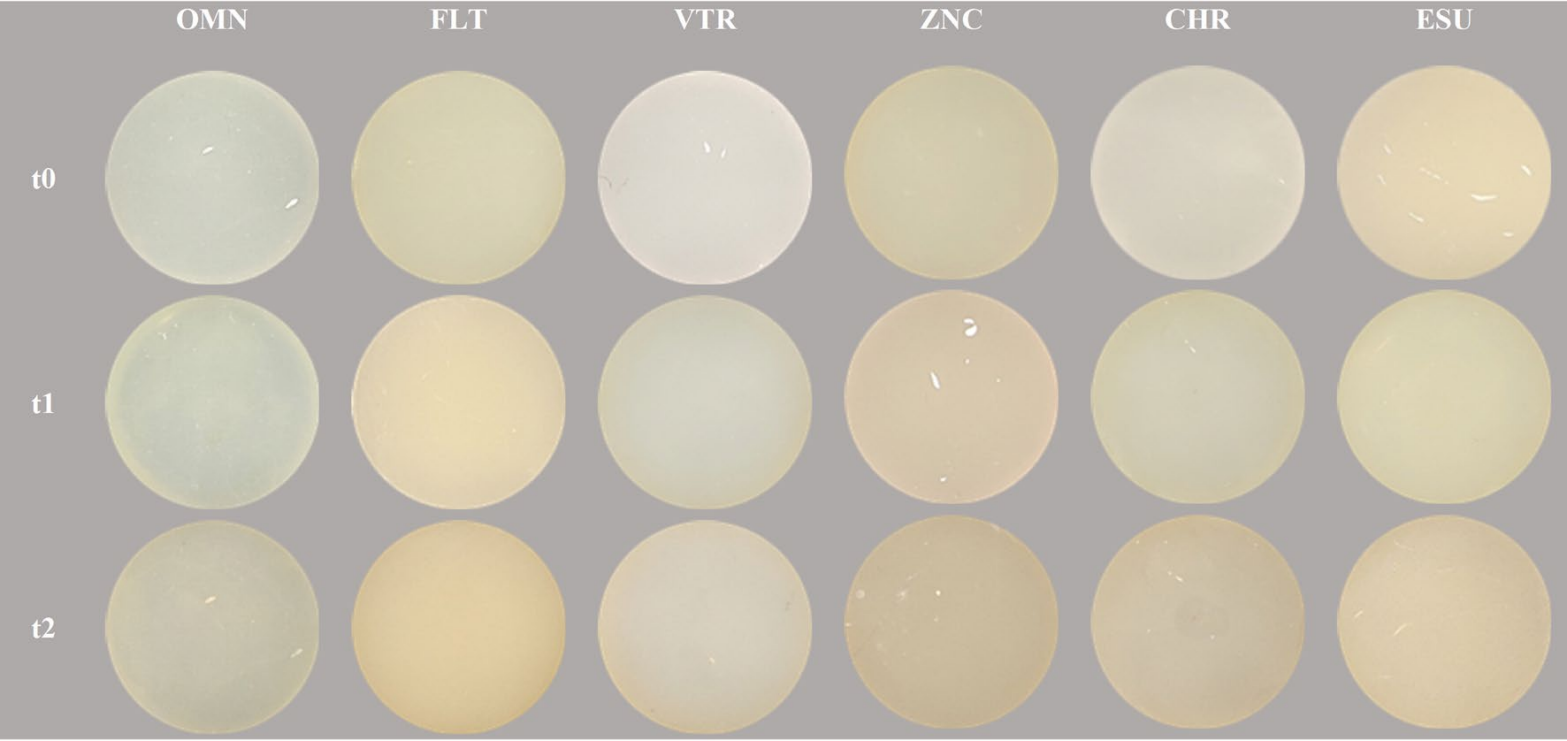
Representative cross-polarized photograph of specimens from each group

The second method involved capturing CP photographs using a Smile-Lite MDP attached to a Xiaomi 11T Pro smartphone (Xiaomi Corporation, China) in super macro mode. A tripod was used to ensure consistency in photography, maintaining a fixed distance of 25 cm from the specimens. Photographs were taken under standardized lighting conditions in the same room, with the camera’s white balance set to 5500 K and the exposure adjusted using an 18% grey card (L*=50, a*=0, b*=0) (Fig. 2). The CP photographs were then processed using Adobe Photoshop Lightroom Classic software, and the L*, a*, and b* values were extracted from the images. Measurements were taken at three different points on each specimen, and the average values were calculated.

### Color differences

The color changes between the time points were calculated and expressed as ΔE_1_ (color change between t_0_ and t_1_) and ΔE_2_ (color change between t_1_ and t_2_). The ΔE_00_ color difference formula was applied to quantify these differences, accounting for variations in lightness (ΔL), chroma (ΔC), and hue (ΔH). The following equation was used for the calculation: [25]

$$\Delta E00 = \sqrt {\begin{aligned}&{{\left( {{{\Delta L} \over {{K_L} + }}} \right)}^2} + {{\left( {{{\Delta C} \over {{K_C} + C}}} \right)}^2} + {{\left( {{{\Delta H} \over {{K_H} + H}}} \right)}^2} \cr&\quad+ \left( {{R_T}\left( {{{\Delta C} \over {{K_C} + C}}} \right)\left( {{{\Delta H} \over {{K_H} + H}}} \right)} \right)\end{aligned}} $$

In this formula, ΔL, ΔC, and ΔH represent the differences in lightness, chroma, and hue between time points. SL, SC, and SH are weighting functions that adjust for variations in the L*, C*, and H* coordinates. The parameters KL, KC, and KH were set to 1, following standard experimental conditions. The rotation term (RT) accounts for the interaction between chroma and hue differences, particularly in the blue region of the color space [25]. 

All ΔE values were calculated using an online ΔE calculator (http://www.colormine.org/delta-e-calculator/Cie2000), ensuring consistent application of the ΔE_00_ formula.

### Staining procedure

The coffee solution was prepared by dissolving 3.6 g of coffee in 300 ml of boiling water at 100 °C, following the manufacturer’s recommendations [26]. The solution was allowed to cool to 37 °C before use. Each specimen was individually immersed in 1.5 ml Eppendorf tubes filled with the coffee solution. The tubes were placed in an incubator at 37 °C to simulate intraoral conditions, where the specimens were stored for 8 h per day for 10 days (t_1_) and for 12 days to simulate 1 year of aging (t_2_) [10, 26]. 

To ensure consistent exposure to the staining solution and prevent contamination, the specimens were rotated and immersed in fresh solution daily. This process ensured uniform contact between the specimens and the staining solution, minimizing the risk of bacterial or fungal growth [26]. 

### Brushing procedure

The specimens removed from the staining solutions were subjected to a brushing simulation using a brushing simulator (MF-100, Mod Dental, Esetron Smart Robotechnologies, Turkey). For the simulation, a toothbrush (Colgate Extra Clean 1 + 1, Colgate Palmolive, USA) and toothpaste (Sensodyne Pronamel Repair, Haleon, United Kingdom) with a relative dentin abrasivity (RDA) of 75, diluted 1:3 by volume, were utilized.

The specimens underwent 300 brushing cycles to simulate 10 days of brushing (t_1_) and 10,000 cycles to replicate 1 year of brushing (t_2_), under a load of 250 g. The brushing was performed in a rotational motion with a movement diameter of 19 mm and a speed of 40 mm/sec. After each set of brushing cycles, the brush heads and toothpaste slurry were replaced for each specimen. Following the simulation, the specimens were rinsed under running water to remove any debris [11, 27, 28]. 

### Thermal cycling procedure

After the brushing simulation, the specimens were subjected to thermal cycling using a thermal cycler (MTE-101, Mod Dental, Esetron Smart Robotechnologies, Turkey). Thermal cycling was conducted by aging the specimens through 300 cycles for 10 days (t_1_) and 10,000 cycles for 1 year (t_2_), simulating long-term intraoral temperature fluctuations. The specimens were alternately immersed in hot (55 °C) and cold (5 °C) water baths, with a dwell time of 30 s in each bath and a transfer time of 10 s between baths. The temperature variance was maintained at ± 2 °C throughout the procedure to ensure consistent thermal stress. This process replicates the thermal challenges faced by dental materials in clinical conditions due to the consumption of hot and cold foods and beverages [12]. 

### AFM and SEM examination

For surface analysis, one random specimen from each group was selected for evaluation using an Atomic Force Microscope (AFM) (Park Systems XE 100 Atomic Force Microscope, Korea) at time points t_0_, t_1_, and t_2_. The AFM was operated in contact mode with a scan size of 10 × 10 μm and a scan speed of 4000 μm/sec. This method provided 3D surface images, allowing for detailed visualization of surface topography changes over time due to the experimental conditions.

Additionally, one specimen from each group was analyzed using a Scanning Electron Microscope (SEM) (Regulus 8230 FE-SEM, Hitachi High Tech Corporation, Japan) at the same time points (t_0_, t_1_, and t_2_). Prior to SEM examination, all specimens were surface coated with a 4 nm layer of gold and palladium to enhance surface conductivity. SEM images were captured under 5000x magnification, at an operating voltage of 3 kV. These high-resolution images provided detailed insights into the surface morphology of the composites at various stages of the study.

After the SEM examination, the gold sputter coating was carefully removed using alcohol to allow the same specimen to undergo further testing. Following the staining and brushing procedures, the specimens were reanalyzed to observe surface changes induced by these treatments. This ensured that the surface remained clean for mechanical testing and provided a clear comparison of surface morphology before and after the treatments [27]. 

### Statistical analysis

The data were analyzed using IBM SPSS software (V25.0, IBM Corp, Armonk, NY, USA). The normality of the data distribution was tested with the Shapiro-Wilk test. For parameters that followed a normal distribution, comparisons between groups were made using One-Way Analysis of Variance (ANOVA), followed by multiple comparisons using Tukey’s Honestly Significant Difference (HSD) or Tamhane tests. For non-normally distributed parameters, the Kruskal-Wallis test was used, with multiple comparisons carried out using the Dunn test.

When comparing dependent variables at two time points, paired t-tests were used for normally distributed data, while the Wilcoxon test was applied for non-normally distributed data. For comparisons at three time points, repeated measures ANOVA was performed for normally distributed data, with Bonferroni correction applied for multiple comparisons. For non-normally distributed data, the Friedman test was conducted, followed by the Dunn test for multiple comparisons.

The agreement between methods was evaluated using the Intra-Class Correlation Coefficient (ICC). Results were expressed as mean ± standard deviation for normally distributed data and as median (minimum– maximum) for non-normally distributed data. The significance level of *p* < 0.05 was considered statistically significant.

## Results

The study demonstrated significant color changes and surface alterations in all composite groups over time. Figure 2 shows representative specimens of single-shade and multi-shade composite resins at baseline (t_0_), after 10 days of staining, brushing and aging (t_1_), and after 1 year of staining, brushing and aging (t_2_).

### ΔE measurements (spectrophotometer and CP photograph)

#### Spectrophotometer ΔE values

Significant differences in ΔE_1_ values were found among the groups (*p* < 0.001). The lowest mean ΔE_1_ value was observed in the FLT group (2.85), while the highest was in the CHR group (7.71). Similarly, ΔE_2_ values differed significantly across groups (*p* < 0.001), with ZNC showing the lowest median value (5.7) and CHR the highest (9.95). Significant within-group differences were also observed for both ΔE_1_ and ΔE_2_ (*p* = 0.005).

#### CP photograph ΔE values

ΔE_1_ values measured using CP photographs also demonstrated significant differences among the groups (*p* < 0.001), with OMN showing the lowest mean value (1.33) and CHR the highest (3.07). ΔE_2_ values followed a similar trend, with FLT showing the lowest median (3.39) and CHR the highest (5.25). Significant within-group differences for both ΔE_1_ and ΔE_2_ were also detected (*p* = 0.005) (Table 2).

**Table 2:** Comparison of ΔE values measured by different methods for groups over time

	FLT	OMN	VTR	ZNC	CHR	ESU	Test stat	*p* value
Spec ΔE_1_	2.85 ± 0.86^b^	3.22 ± 1.34^b^	7.06 ± 2.18^a^	2.87 ± 1.15^b^	7.71 ± 2.04^a^	7.01 ± 1.99^a^	19.157	< 0.001*
	2.54 (1.94–4.23)	2.82 (1.83–5.92)	7.82 (4.1–9.58)	2.79 (0.96–4.82)	7.17 (4.4–10.22)	6.64 (4.85–10.56)		
Spec ΔE_2_	5.87 ± 1.41	9.62 ± 1.39	8.2 ± 1.72	5.64 ± 1.96	9.68 ± 1.38	8.4 ± 1.81	31.052	< 0.001**
	6.35 (2.4–7.09)^b^	9.48 (7.45–11.99)^a^	8.82 (5.74–10.25)^ab^	5.7 (2.84–8.08)^b^	9.95 (6.93–11.21)^a^	8.04 (6–11.22)^ab^		
Test stat.	-2.803	-2.803	-2.499	-2.803	-2.805	-2.803		
P***	**0.005**	**0.005**	**0.012**	**0.005**	**0.005**	**0.005**		
CP ΔE_1_	2.94 ± 1.14^bc^	1.33 ± 0.56^a^	3.71 ± 0.7^c^	2.46 ± 0.91^b^	3.07 ± 0.75^bc^	2.88 ± 0.43^bc^	10.37	< 0.001*
	2.91 (1.62–5.37)	1.2 (0.68–2.55)	3.51 (2.74–4.75)	2.49 (0.7–3.74)	3.35 (1.89–4.06)	2.78 (2.3–3.69)		
CP ΔE_2_	4.63 ± 2.59	4.39 ± 0.41	5.01 ± 0.47	3.85 ± 0.65	5.24 ± 0.56	3.53 ± 0.55	29.293	< 0.001**
	3.39 (2.58–9.52)^ac^	4.55 (3.73–4.8)^ab^	4.95 (4.07–5.65)^bc^	3.64 (3.09–5.39)^a^	5.25 (3.98–5.89)^b^	3.61 (2.68–4.43)^a^		
Test stat.	-2.803	-2.803	-2.803	-2.599	-2.803	-2.803		
P***	**0.005**	**0.005**	**0.005**	**0.009**	**0.005**	**0.005**		

*One-way ANOVA; **Kruskal-Wallis Test; ***Wilcoxon Test; Mean ± standard deviation; Median (minimum– maximum); a-c: There is no difference between groups with the same letter

### L*, a*, b* values across time points (spectrophotometer and CP photograph)

#### Spectrophotometer L* values

Significant differences in L* values were observed across all time points in the OMN, CHR, and ESU groups (*p* < 0.001), with t_0_ showing significant differences from both t_1_ and t_2_. In the FLT group, significant differences were found between t_0_ and t_2_. The VTR and ZNC groups also showed significant differences, with t_0_ and t_2_ values differing significantly.

#### CP photograph L* values

For L* values measured by CP photographs, significant differences were observed in the OMN, VTR, ZNC, CHR, and ESU groups (*p* < 0.001), with t_2_ values differing significantly from t_0_ and t_1_ in most groups. However, in the FLT group, no significant differences were observed between time points (Table 3).

**Table 3:** Comparison of L values measured by different methods for groups over time

	FLT	OMN	VTR	ZNC	CHR	ESU
t_0_ Spec L*	77.08 ± 1.05	73.57 ± 1.83 ^a^	79.8 ± 1.08	70.46 ± 5.58	70.01 ± 1.18 ^a^	74.34 ± 0.74 ^a^
	77.2 (74.46–78.03) ^a^	73.58 (71.2–76.86)	79.75 (77.3–81.33) ^a^	68.97 (66.13–86) ^a^	70.15 (67.96–71.63)	74.28 (72.96–75.63)
t_1_ Spec L*	74.77 ± 1.02	69.57 ± 0.98 ^b^	70.67 ± 2.68	65.76 ± 1.6	61.73 ± 1.93 ^b^	65.85 ± 3.34 ^b^
	74.58 (73.76–77.43) ^ab^	69.38 (68.5–71.9)	69.28 (67.36–74.23) ^b^	66.32 (62.6–68.5) ^ab^	61.33 (59.63–65.26)	65.9 (61.06–72.36)
t_2_ Spec L*	69.78 ± 2.02	64.11 ± 0.98 ^c^	68.94 ± 2.54	64.29 ± 3.07	60.48 ± 1.46 ^b^	64.58 ± 2.31 ^b^
	69.07 (68–75.2) ^b^	64.09 (62.43–65.86)	68.5 (65.73–73.82) ^b^	64.56 (60.2–68) ^b^	60.08 (58.83–63.26)	64.58 (61.43–67.76)
Test stat.	16.200	149.234	16.800	10.400	90.646	82.526
P value	< 0.001**	< 0.001*	< 0.001**	0.006**	< 0.001*	< 0.001*
t_0_ CP L*	86.86 ± 0.42	81.83 ± 0.55	86.55 ± 0.66 ^a^	81.4 ± 0.74 ^a^	84.24 ± 0.6	86.11 ± 0.37 ^a^
	87.1 (86.13–87.2) ^b^	81.73 (80.93–82.7) ^a^	86.63 (85.56–87.76)	81.45 (80.16–82.43)	84.23 (83.56–85.26) ^a^	86.15 (85.56–86.76)
t_1_ CP L*	85.32 ± 0.75	81.99 ± 0.6	84.11 ± 0.64 ^b^	80.55 ± 0.52 ^b^	82.23 ± 0.54	84.61 ± 0.43 ^b^
	85.5 (83.9–86.26) ^ab^	81.92 (81.2–82.86) ^a^	83.95 (83.13–85.56)	80.43 (79.83–81.6)	82.02 (81.7–83.03) ^ab^	84.6 (84.03–85.36)
t_2_ CP L*	82.33 ± 1.01	78.98 ± 0.89	82.02 ± 0.85 ^c^	76.42 ± 0.72 ^c^	78.41 ± 0.61	81.25 ± 0.74 ^c^
	82.72 (80.33–83.72) ^a^	78.81 (78.03–80.16) ^b^	82.01 (80.36–83.03)	76.38 (75.03–77.4)	78.32 (77.46–79.16) ^b^	81.52 (79.96–82.16)
Test stat.	20.000	15.200	88.722	180.516	20.000	261.674
P Value	< 0.001**	0.001**	< 0.001*	< 0.001*	< 0.001**	< 0.001*

*Repeated Measures Analysis of Variance ** Friedman Test; Mean ± standard deviation; Median (minimum– maximum); a-c: There is no difference between times that share the same letter

#### Spectrophotometer a* values

Significant changes in a* values were observed across time points in the OMN, CHR, and ESU groups (*p* < 0.001), with significant differences across all time points (t_0_, t_1_, and t_2_). In the ZNC group, a values at t_2_ differed significantly from those at t_0_ and t_1_. In the FLT group, significant differences were found between t_1_ and t_2_, but not between other time points.

#### CP photograph a* values

For a* values measured by CP photographs, significant differences were noted in the OMN, VTR, ZNC, CHR, and ESU groups (*p* < 0.001). In the FLT group, a* values showed significant differences between t_1_ and t_2_, while no significant differences were found between t_0_ and the other time points (Table 4).

**Table 4:** Comparison of a* values measured by different methods for groups over time

	FLT	OMN	VTR	ZNC	CHR	ESU
t_0_ Spec a*	0.85 ± 0.19	-5.24 ± 0.28 ^a^	-4.87 ± 0.12 ^a^	-2.18 ± 0.28 ^a^	-3.92 ± 0.23 ^a^	-0.56 ± 0.15
	0.82 (0.63–1.26) ^b^	-5.23 (-5.6 - -4.86)	-4.9 (-5.03 - -4.63)	-2.12 (-2.83 - -1.9)	-3.95 (-4.3 - -3.46)	-0.58 (-0.73 - -0.33) ^b^
t_1_ Spec a*	-0.25 ± 0.24	-4.28 ± 0.23 ^b^	-3.85 ± 0.43 ^b^	-2.19 ± 0.21 ^a^	-1.95 ± 0.68 ^b^	0.66 ± 0.45
	-0.22 (-0.8–0.06) ^a^	-4.36 (-4.7 - -4)	-3.9 (-4.4 - -3.13)	-2.2 (-2.56 - -1.76)	-1.78 (-2.96 - -1.1)	0.48 (0.3–1.76) ^a^
t_2_ Spec a*	1.54 ± 0.51	-1.68 ± 0.22 ^c^	-3.32 ± 0.85 ^b^	-0.57 ± 0.73 ^b^	-0.84 ± 0.38 ^c^	1.42 ± 0.81
	1.4 (1.16–2.91) ^b^	-1.71 (-1.96 - -1.4)	-3.54 (-4.36 - -1.86)	-0.55 (-1.53–0.46)	-0.82 (-1.63 - -0.33)	1.41 (0.06–2.43) ^a^
Test stat.	18.200	810.405	16.245	36.188	171.050	16.800
P value	< 0.001**	< 0.001*	0.002*	< 0.001*	< 0.001*	< 0.001**
t_0_ CP a*	1.4 ± 0.2	-2.81 ± 0.46	0.48 ± 0.43 ^a^	1.74 ± 0.51 ^a^	0.19 ± 0.3 ^a^	1.34 ± 0.26 ^a^
	1.35 (1.23–1.9) ^ab^	-2.73 (-3.53 - -2.1) ^a^	0.37 (0.03–1.4)	1.7 (1.1–2.66)	0.12 (-0.16–0.83)	1.32 (0.93–1.83)
t_1_ CP a*	-0.51 ± 0.39	-2.76 ± 0.62	-1.36 ± 0.16 ^b^	-0.21 ± 0.33 ^b^	-1.12 ± 0.33 ^b^	-0.84 ± 0.14 ^b^
	-0.55 (-1.16–0.13) ^a^	-2.76 (-3.6 - -1.4) ^a^	-1.45 (-1.56 - -1.13)	-0.2 (-0.63–0.43)	-1.18 (-1.53 - -0.63)	-0.82 (-1.03 - -0.63)
t_2_ CP a*	2.53 ± 0.16	-1.31 ± 0.35	0.6 ± 0.26 ^a^	2.44 ± 0.2 ^c^	1.45 ± 0.55 ^c^	2.29 ± 0.36 ^c^
	2.53 (2.26–2.8) ^b^	-1.29 (-2.16 - -1) ^b^	0.62 (0.16–1.06)	2.42 (2.06–2.83)	1.33 (0.7–2.5)	2.36 (1.6–2.9)
Test stat.	20.000	15.200	167.070	186.303	89.987	344.237
P value	< 0.001**	0.001**	< 0.001*	< 0.001*	< 0.001*	< 0.001*

* Repeated Measures Analysis of Variance ** Friedman Test; Mean ± standard deviation; Median (minimum– maximum); a-c: There is no difference between times that share the same letter

#### Spectrophotometer b* values

Significant differences in b* values were observed across all time points in the OMN, CHR, and ESU groups (*p* < 0.001). In these groups, b* values at t_2_ differed significantly from both t_0_ and t_1_. In the FLT group, significant differences were observed between t_1_ and t_2_, but not between other time points. In the VTR and ZNC groups, significant differences were found between t_0_ and t_2_.

#### CP photograph b* values

For b* values measured by CP photographs, significant differences were observed in the OMN, VTR, ZNC, CHR, and ESU groups (*p* < 0.001), with t_2_ showing differences from t_0_ and t_1_ in most groups. In the FLT group, significant differences were found only between t_1_ and t_2_ (Table 5).

**Table 5:** Comparison of b* values measured by different methods for groups over time

	FLT	OMN	VTR	ZNC	CHR	ESU
t_0_ Spec b*	20.91 ± 0.83	5.18 ± 4.23	4.38 ± 0.26 ^a^	8.25 ± 0.93 ^a^	6.76 ± 0.58 ^a^	16.19 ± 0.42
	20.55 (20.13–22.5) ^a^	6.58 (-6.76–7.1) ^b^	4.28 (4–4.8)	8.47 (6.06–9.26)	6.83 (5.9–7.56)	16.16 (15.66–16.83) ^b^
t_1_ Spec b*	18.63 ± 1.34	7.37 ± 0.5	6.53 ± 1.54 ^b^	8.22 ± 0.65 ^a^	10.03 ± 1.15 ^b^	17.68 ± 2.49
	19.05 (15.83–20.16) ^a^	7.3 (6.7–8.16) ^ab^	6.35 (4.6–8.93)	8.22 (7.06–9.1)	10.05 (8.5–11.76)	17.25 (14.86–23.96) ^ab^
t_2_ Spec b*	24.57 ± 1.72	12.1 ± 0.44	7.49 ± 1.87 ^b^	12.21 ± 1.09 ^b^	11.8 ± 0.5 ^c^	19.18 ± 1.87
	25.07 (21.26–26.43) ^b^	12.22 (11.3–12.6) ^a^	7.57 (5.3–11)	12.31 (10.5–14.23)	11.75 (11.1–12.63)	19.27 (15.56–22.3) ^a^
Test stat.	18.200	20.000	10.132	55.046	160.053	8.600
P value	< 0.001**	< 0.001**	0.007*	< 0.001*	< 0.001*	0.014**
t_0_ CP b*	20.81 ± 0.6 ^a^	7.47 ± 0.42 ^a^	5.28 ± 0.43 ^a^	15.1 ± 0.52 ^a^	9.41 ± 0.59 ^a^	17.58 ± 0.51
	20.73 (20.03–21.93)	7.38 (6.93–8.2)	5.45 (4.66–5.76)	15.17 (14.13–16.13)	9.32 (8.6 − 0.46)	17.51 (17.03–18.86) ^ab^
t_1_ CP b*	17.85 ± 1.06 ^b^	8.29 ± 0.84 ^a^	8.01 ± 0.54 ^b^	14.8 ± 0.6 ^a^	12.43 ± 0.89 ^b^	16.97 ± 0.81
	17.98 (15.26–19)	8.33 (6.86–9.4)	7.88 (7.33–9.2)	14.83 (13.6–15.5)	12.36 (10.93–13.63)	16.9 (15.63–18.56) ^a^
t_2_ CP b*	21.41 ± 0.66 ^a^	11.6 ± 0.54 ^b^	10.5 ± 0.88 ^c^	17.5 ± 0.74 ^b^	13.92 ± 0.58 ^c^	18.51 ± 1.53
	21.27 (20.63–22.66)	11.6 (10.7–12.4)	10.43 (9.13–11.83)	17.33 (16.7–19.23)	13.96 (12.9–14.7)	17.93 (16.86–21.33) ^b^
Test stat.	55.658	147.510	167.931	95.560	119.248	10.400
P value	< 0.001*	< 0.001*	< 0.001*	< 0.001*	< 0.001*	0.006**

* Repeated Measures Analysis of Variance ** Friedman Test; Mean ± standard deviation; Median (minimum– maximum); a-c: There is no difference between times that share the same letter

### Comparison of Agreement between Measurement methods

For the ΔE_1_ and ΔE_2_ parameters, there was a statistically weak agreement between the spectrophotometer and photographic methods. A statistically moderate agreement was observed for the L* values at t_0_, t_1_, and t_2_, as well as for the a* value at t_1_ (*p* < 0.001). Statistically good agreement was found for the a* values at t_0_ and t_2_, and for the b* values at t_1_ and t_2_ (*p* < 0.001). The b* value at t_0_ showed statistically excellent agreement between the methods (*p* < 0.001) (Table 6).

**Table 6:** Investigation of the agreement between spectrophotometer and CP photography methods

	ICC (%95 CI)	*P* value
ΔE_1_	0.379 (-0.039–0.629)	**0.035**
ΔE_2_	0.068 (-0.56–0.443)	0.393
t_0_ L*	0.657 (0.426–0.795)	**< 0.001**
t_1_ L*	0.5 (0.164–0.702)	**0.004**
t_2_ L*	0.653 (0.42–0.793)	**< 0.001**
t_0_ a*	0.785 (0.639–0.871)	**< 0.001**
t_1_ a*	0.663 (0.436–0.799)	**< 0.001**
t_2_ a*	0.76 (0.598–0.857)	**< 0.001**
t_0_ b*	0.942 (0.904–0.966)	**< 0.001**
t_1_ b*	0.897 (0.828–0.939)	**< 0.001**
t_2_ b*	0.894 (0.823–0.937)	**< 0.001**

Intraclass Correlation Coefficient (95% Confidence Interval)

### AFM results

**Fig. 3:**
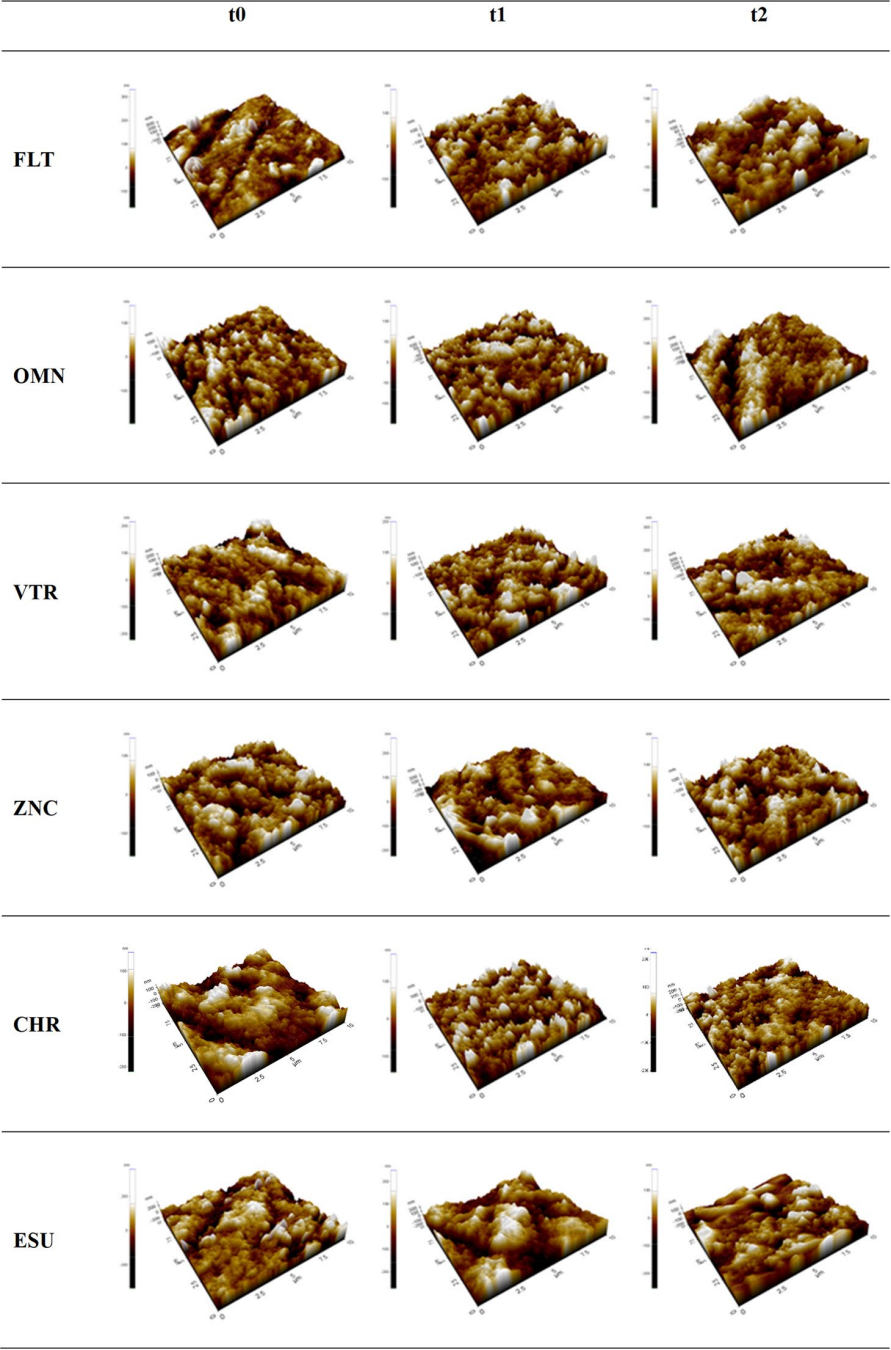
AFM analysis reveals the surface topography of specimens from each group

At t_0_, the surfaces of all composite groups exhibited relatively smooth topographies with evenly distributed peaks and valleys. By t_1_, noticeable surface changes were observed, with the formation of more distinct peaks and deeper valleys, indicating increased surface roughness. At t_2_, further surface degradation was evident, as the valleys became deeper and the peaks more pronounced, suggesting significant surface wear and roughness across all groups. The increase in the number and size of peaks and valleys from t_0_ to t_2_ indicates progressive surface irregularities over time (Fig. 3).

### SEM results

**Fig. 4:**
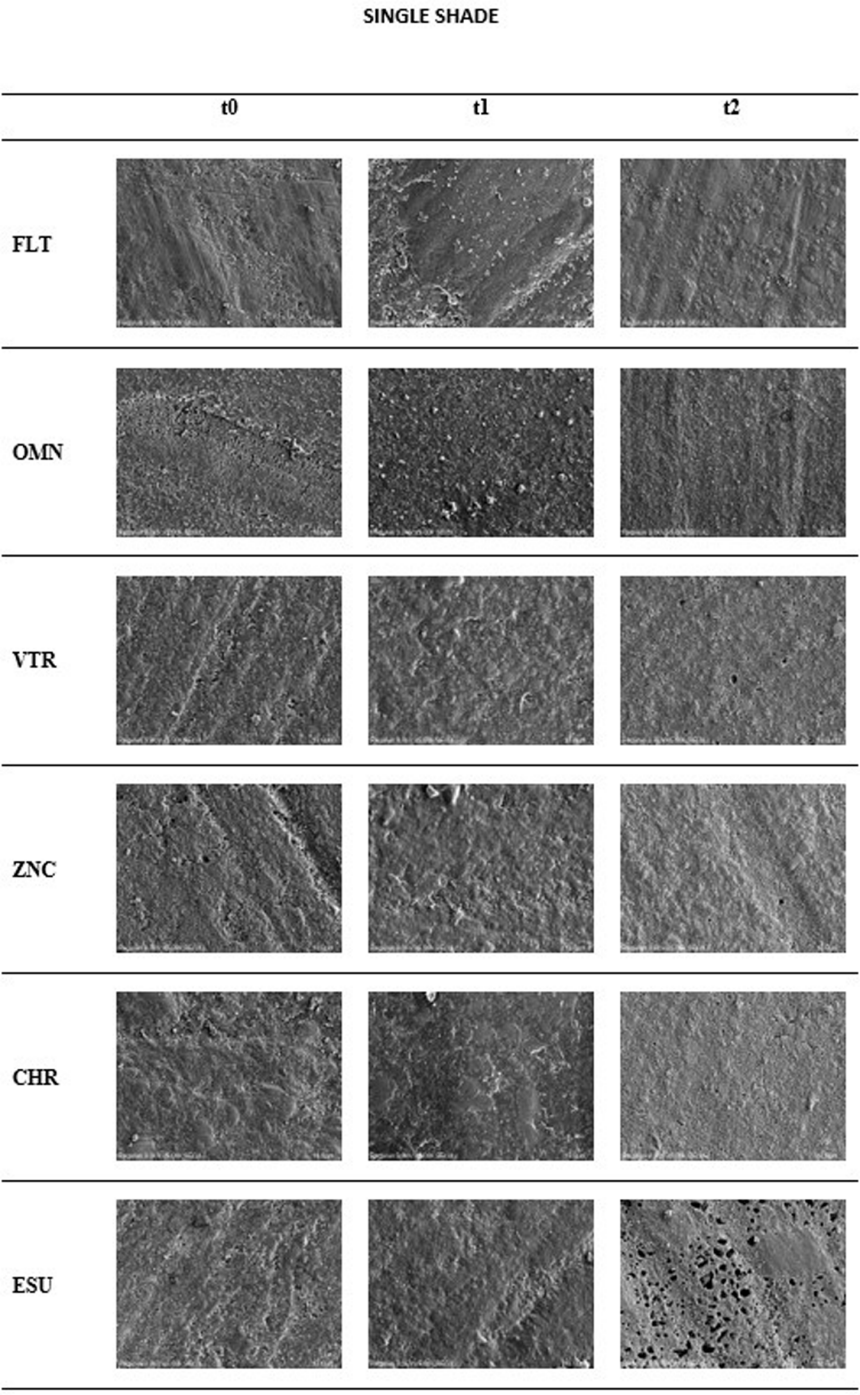
SEM analysis at 5000x magnification illustrates the surface morphology of specimens from each group

At t_0_, the surfaces of all composite groups appeared relatively smooth, with minor surface irregularities. By t_1_, surface degradation became more apparent, with the emergence of cracks and rough areas. At t_2_, further surface deterioration was observed, characterized by more pronounced cracks and the appearance of voids (Fig. 4).

## Discussion

The lack of a definitive understanding of the color stability of single-shade composite resins in clinical use highlights the importance of in vitro studies, such as this one, to provide initial insight into their long-term aesthetic durability. This study evaluated the color stability of single- shade composite resins in response to external factors, including staining, brushing and thermal cycling. The results showed significant color changes in all composite groups, as measured consistently by both spectrophotometer and CP photography. Notably, the CHR group exhibited the highest ΔE_1_ and ΔE_2_ values, indicating the greatest degree of color change. For ΔE_1_, the lowest value was observed in the FLT group using spectrophotometry and in the OMN group using CP photography. For ΔE_2_, the lowest values were observed in the ZNC group by spectrophotometry and in the FLT group by CP photography. A positive correlation was found between spectrophotometric and CP photographic measurements for all parameters. These results underscore the detrimental effect of external factors on the aesthetic performance of composite resins and the critical importance of maintaining shade stability for the long-term success of these materials.

The first hypothesis of the study was that the color changes resulting from these procedures would not reach statistical significance. However, this hypothesis was rejected as significant differences in ΔE_1_ and ΔE_2_ were observed across all groups (*p* < 0.001). These results suggest that single-shade composites are particularly susceptible to shade changes over time, especially due to staining and brushing, which are common in clinical practice.

The second hypothesis was that no significant correlation would be found between spectrophotometer and CP photography measurements. In contrast, a correlation was found, especially for L*, a*, and b* values** at specific time points (t_0_, t_1_, t_2_) (*p* < 0.001), although the correlation was weaker for ΔE_1_ and ΔE_2_. While spectrophotometry provides more accurate measurements, CP photography with a smartphone is a practical alternative for clinical use. Therefore, this hypothesis was also rejected.

Spectrophotometer and CP photography each have specific advantages in evaluating color stability. Spectrophotometers provide precise, objective measurements, making them particularly suitable for research, while CP photography offers a practical, accessible option for clinical use [13, 20, 22]. In particular, specimen images taken with CP photography allow for targeted color assessment in specific areas, enabling the detection of color variations within the same sample [29, 30]. Although image-based color measurement is not universally standardized, studies have shown that reliable results comparable to spectrophotometer readings can be achieved by standardizing image capture and using a neutral gray card for white balance adjustment [30, 31]. Traditionally, DSLR cameras have been preferred for such measurements due to their superior sensor quality, lens options, and manual control. However, smartphones are becoming increasingly popular for color evaluation due to their accessibility, portability, and ease of use. In this study, CP photographs were taken using a Smile-Lite MDP with a smartphone and a gray card to obtain accurate color images without requiring specialized expertise, alongside spectrophotometry as the other method for color assessment [32]. 

In a study compared the color matching of single-shade and multi-shade composites specifically OMN, ZNC, VTR, CHR, Clearfil Majesty ES-2 Universal (Kuraray Noritake, Japan), and Estelite Asteria (Tokuyama Dental Corporation, Japan) using smartphone photography with Smile Lite MDP and visual shade selection. Results indicated that single-shade composites did not show superior color adaptation compared to multi-shade composites [33]. In another study evaluating color matching in single-shade composites found CHR to have superior color matching compared to VTR. Using a spectrophotometer and a smartphone with CP filters on the Smile Lite MDP device, with white balance adjusted via Adobe Photoshop Lightroom Classic, a positive correlation was observed between spectrophotometer and image-based measurements for the L*, a*, and b* coordinates [34]. Comparable findings were observed in a similar study that used a DSLR camera in place of a smartphone [30]. According to other study evaluating the color stability of resin-based restorative materials before and after polymerization using spectrophotometer and CP photography with Adobe Photoshop CC, a positive correlation was found between the obtained ΔE values [20]. In the present study, similarly, the two methods were compared to determine their effectiveness in detecting color stability in single-shade composites. For all parameters, a positive correlation was found between spectrophotometer and CP photography, suggesting that the methods may vary in sensitivity and accuracy under certain conditions.

Previous studies using spectrophotometer and CP photography have primarily focused on the color matching capabilities of single-shade composites, assessing how well they blend with natural teeth or other composite materials upon initial placement [30, 33, 34]. However, these studies often lack simulation of intraoral conditions such as thermal cycling, staining, and brushing, which are essential for evaluating long-term color stability. Replicating these conditions is critical, as factors like brushing can both remove surface pigments and cause contour loss through abrasive wear, directly impacting color stability [11, 27, 28]. Staining agents, especially coffee, produce varying degrees of discoloration, and thermal cycling can exacerbate color changes by inducing microcracks and weakening the resin matrix, which reduces structural durability [10, 12, 35]. Given these considerations, the present study focused on color stability rather than initial color matching, incorporating simulated intraoral conditions to provide a more comprehensive assessment of how single-shade composites maintain aesthetic integrity. Although clinical evidence is limited, this in vitro study offers foundational data on the potential color stability of single-shade composite resins under conditions mimicking the oral environment.

The CHR group exhibited the most significant color changes (ΔE_1_ and ΔE_2_) among the tested materials, which may be attributed to the presence of larger filler particles. These particles can create a rougher surface texture, increasing susceptibility to staining agents, and lead to greater water absorption [36]. Water molecules penetrating the filler-matrix interface may cause hydrolytic degradation, further contributing to discoloration [37]. These findings are consistent with previous research highlighting the role of filler size and surface characteristics in the discoloration of composite resins during aging and exposure to staining agents [10, 36]. The differences in ΔE_1_ values observed among the composite groups can be attributed to variations in their composition and filler properties. The FLT group, a multi-shade composite resin, exhibited the lowest ΔE_1_ value, which may be related to its filler particle size, distribution, and resin matrix composition [1]. Multi-shade composites such as FLT are often designed with a balance of translucency and opacity that allows for better blending with the surrounding tooth structure while maintaining color stability [37]. 

ΔE values were calculated using the CIEDE2000 formula, which offers improved sensitivity over the CIELAB formula (Commission Internationale de l’Eclairage Lab*) in detecting color differences. To enhance alignment with visual assessments, the ISO (International Standards Organization) and CIE (Commission Internationale de l’Eclairage) currently recommend the CIEDE2000 formula, derived from the CIELAB color space, for calculating total color differences. For this reason, this formula was also used in the present study [10]. 

In the dental literature, a ΔE_00_ value of 1.8 is generally accepted as the perceptibility threshold and 2.7 as the acceptability threshold [38–40]. In our study, only the OMN group showed a ΔE_1_ value (1.33 ± 0.56) below the perceptual threshold, indicating no clinically visible color change at this stage. This suggests that OMN has strong short-term color stability. However, the ΔE_2_ values for OMN (9.62 ± 1.39) were significantly above both thresholds, indicating significant discoloration over time under simulated intraoral conditions. On the other hand, the CHR group exhibited the highest shade changes in both ΔE_1_ (7.71 ± 2.04) and ΔE_2_ (9.68 ± 1.38) values, significantly above the acceptability thresholds. This pronounced discoloration can be attributed to larger filler particle sizes, which can create a rougher surface, making the material more susceptible to staining agents. In addition, larger filler particles can increase water absorption at the filler-matrix interface, leading to hydrolytic degradation and further discoloration. Previous studies have shown that water absorption can contribute to matrix degradation, thereby accelerating color changes in composite resins [12, 36, 37]. In contrast, the FLT group showed relatively less color change in both ΔE_1_ (2.85 ± 0.86) and ΔE_2_ (5.87 ± 1.41). While these values are above the perceptual threshold, they remain closer to the acceptability threshold compared to single shade composites. This suggests that multi-shade composites such as FLT may offer better long-term shade stability. This may be due to the layered structure of FLT with different opacities and a more uniform filler distribution, which contributes to improved color retention [37]. These findings suggest that while single-shade composites may provide excellent initial color matching, they are more prone to discoloration over time, especially when exposed to staining agents and thermal cycling. The integration of perceptibility and acceptability thresholds in this discussion offers a clearer understanding of the clinical relevance of our findings. Further long-term clinical studies are necessary to validate the color stability of single-shade composites under real intraoral conditions.

This study offers insights into the long-term color stability of single-shade composite resins when subjected to simulated intraoral conditions. The findings underscore the susceptibility of these materials to discoloration and underscore the importance of material selection for the maintenance of aesthetic outcomes in restorative dentistry. Furthermore, CP photography has the potential to serve as a practical and accessible tool for clinical color assessment, offering reliable results that are comparable to those obtained through spectrophotometry.

By systematically evaluating color stability under controlled conditions, this study aims to strengthen the scientific basis for assessing single-shade composite resins in aesthetic applications.

### Limitations

It is important to consider the limitations of this study when interpreting the results. First, as an in vitro study, it does not fully replicate all intraoral conditions, such as interactions with oral flora, saliva, and continuous temperature and pH fluctuations. While brushing, staining, and thermal cycling simulations offer valuable insights, they may not fully capture the complexity of the oral cavity. Moreover, the findings are specific to the single-shade and multi-shade composite materials tested in this study, and generalizing these results to other brands or types of composite materials should be approached with caution. The results are specific to the materials tested, and additional studies with broader material ranges and in vivo conditions may provide further insight into clinical applications. Another limitation of this study is the use of an intraoral spectrophotometer under in vitro conditions. Although intraoral spectrophotometers are designed for clinical use, they may not provide completely reliable results when used in an in vitro setting. Comparing CP photography with such devices could introduce a potential bias in the results. This limitation underscores the need for cautious interpretation of results, as previously highlighted in the literature [41, 42]. 

## Conclusions

This study demonstrated significant color changes in single shade composite resins when exposed to simulated intraoral conditions such as brushing, staining, and thermal cycling. CHR exhibited the highest color change values (ΔE_1_ and ΔE_2_), which were attributed to its filler properties, including larger particle size. The use of intra-oral spectrophotometers and CP photography was found to be comparable in evaluating color stability within the context of this study, considering the limitations of intra-oral spectrophotometers in in vitro settings. These results provide valuable insights into the color stability of single shade composite resins and their potential aesthetic performance under challenging conditions.

## Data Availability

All data analyzed during this study are included in the article in the form of tables. Raw data supporting the findings of this study are available from the corresponding author upon reasonable request.

## Abbreviations

CP: Cross-polarized
OMN: Omnichroma
Zenchroma: ZNC
VTR: Vittra APS Unique
CHR: Charisma One
ESU: Essentia Universal
FLT: Filtek Z550
CIE: Commission Internationale de l’Eclairage
CIELAB formula: Commission Internationale de l’Eclairage Lab*
ΔE: Color Changes
HSD: Honestly Significant Difference
ANOVA: One-Way Analysis of Variance
SEM: Scanning Electron Microscope
AFM: Atomic Force Microscope
RDA: Relative Dentin Abrasivity
RT: Rotation Term
ΔL: Lightness
ΔC: Chroma
ΔH: Hue
ICC: Intra-Class Correlation Coefficient
